# A national evaluation analysis and expert interview study of real-world data sources for research and healthcare decision-making

**DOI:** 10.1038/s41598-024-59475-9

**Published:** 2024-04-28

**Authors:** Veronika Mikl, Dejan Baltic, Thomas Czypionka, Alexander Degelsegger-Márquez, Nikolaus Forgó, Ghazaleh Gouya-Lechner, Arnold Herzog, Peter Klimek, David Benjamin Lumenta, Bernhard Mraz, Herwig Ostermann, Robert Scharinger, Tanja Stamm, Michael Strassnig, Markus Zeitlinger, Johannes Pleiner-Duxneuner

**Affiliations:** 1 Gesellschaft für Pharmazeutische Medizin E.V. (GPMed), 1210 Vienna, Austria; 2 Roche Austria GmbH, Vienna, Austria; 3 Amgen GmbH, Vienna, Austria; 4 Institut für Höhere Studien – Institute for Advanced Studies (IHS), 1080 Vienna, Austria; 5 Gesundheit Österreich GmbH (GÖG), 1010 Vienna, Austria; 6 Faculty of Law, Department of Innovation and Digitalisation in Law, University of Vienna, 1010 Vienna, Austria; 7 Gouya Insights GmbH & CoKG, 1190 Vienna, Austria; 8 Austrian Medicines and Medical Devices Agency (AGES Medizinmarktaufsicht), 1220 Vienna, Austria; 9 Supply Chain Intelligence Institute Austria (ASCII), 1080 Vienna, Austria; 10 Section for Science of Complex Systems, Center for Medical Statistics, Informatics, and Intelligent Systems, Medical University Vienna, 1090 Vienna, Austria; 11 Complexity Science Hub Vienna, 1080 Vienna, Austria; 12 Research Unit for Digital Surgery, Division of Plastic, Aesthetic and Reconstructive Surgery, Department of Surgery, Medical University of Graz, 8036 Graz, Austria; 13 Novartis Pharma GmbH, Vienna, Austria; 14 Federal Ministry of Social Affairs, Health, Care and Consumer Protection, 1010 Vienna, Austria; 15 Section for Outcomes Research, Center for Medical Statistics, Informatics, and Intelligent Systems, Medical University of Vienna, Medical University Vienna, 1090 Vienna, Austria; 16 Wiener Wissenschafts-, Forschungs- und Technologiefonds (Vienna Science and Technology Fund), 1090 Vienna, Austria; 17 Expertenplattform Plattform Registerforschung, c/o WWTF, 1090 Vienna, Austria; 18 Department of Clinical Pharmacology, Medical University Vienna, 1090 Vienna, Austria

**Keywords:** Real-world data, Real-world evidence, Data quality, Data quality criteria, Health data use, Secondary use of health data, Health data strategy, FAIR data principles, Data quality recommendations, Pharmaceutical research, Healthcare decision-making, Quality criteria for RWD in health care, Gesellschaft für Pharmazeutische Medizin, GPMed, Health care, Health policy

## Abstract

Real-world data (RWD) can provide intel (real-world evidence, RWE) for research and development, as well as policy and regulatory decision-making along the full spectrum of health care. Despite calls from global regulators for international collaborations to integrate RWE into regulatory decision-making and to bridge knowledge gaps, some challenges remain. In this work, we performed an evaluation of Austrian RWD sources using a multilateral query approach, crosschecked against previously published RWD criteria and conducted direct interviews with representative RWD source samples. This article provides an overview of 73 out of 104 RWD sources in a national legislative setting where major attempts are made to enable secondary use of RWD (e.g. law on the organisation of research, "Forschungsorganisationsgesetz"). We were able to detect omnipresent challenges associated with data silos, variable standardisation efforts and governance issues. Our findings suggest a strong need for a national health data strategy and data governance framework, which should inform researchers, as well as policy- and decision-makers, to improve RWD-based research in the healthcare sector to ultimately support actual regulatory decision-making and provide strategic information for governmental health data policies.

## Introduction

Real-world data (RWD) generate evidence for various research, development, policy and regulatory decision-making purposes along the product lifecycles of pharmaceuticals and medical devices. The increasing use^1–4^ of RWD also provides significant possibilities beyond the aforementioned opportunities across the full spectrum of health care, ranging from clinical trial design to the study of medical (mal-)practice^5^ to public health and health policy^6^. To account for the transformative potential of RWD, the European Union has recently passed in addition to existing legislation such as the General Data Protection Regulation (GDPR) and the European Data Governance Act (DGA^7^). Furthermore, the European Commission (EC) proposed a regulation for the European Health Data Space (EHDS^8^) to facilitate, among other aims, the safe and secure use and reuse of health data for better healthcare delivery, research and policy-making. The recent proposal of the EC to revise pharmaceutical legislation also emphasizes the importance of leveraging RWD in healthcare.^9^ However, progress in the digitalisation of health care systems is unevenly distributed across Europe^10^, casting doubts on achieving the ambitious aims of the EHDS. Despite ongoing initiatives like DARWIN EU^11^, calls from global regulators for international collaboration to integrate real-world evidence (RWE) into regulatory decision-making^12^ and to bridge knowledge gaps, some challenges, such as heterogeneity of data sources, linkability/sharing of data, variable quality of data and differing approaches for data access, require more and appropriate attention. In addition to the outlined ongoing changes, the results of previous work^13^ also indicate the necessity for increased transparency regarding the availability of national RWD sources. The checklist in this work^13^ covers important areas such as data management, governance, quality requirements, data privacy, research objectives, data providers, patient population, data elements, and infrastructure. The checklist incorporates the "FAIR Data Principles," which emphasize the importance of making RWD easy to find, access, use, and reuse for secondary purposes and added value. However, the applicability, value, and practicality of the previously published checklist^13^ on quality criteria for RWD sources have not been evaluated yet.

## Research objectives

In this work, a multi-stakeholder group coordinated by the Gesellschaft für Pharmazeutische Medizin (GPMed, Austrian Society for Pharmaceutical Medicine) compiled and classified already used national RWD sources in Austria and made an in-depth assessment of the research readiness of selected datasets. The group reviewed the previously published quality checklist for RWD in pharmaceutical research and regulatory decision-making^13^ in terms of added value and usability in practice. The results and findings intend to emphasise the relevance of RWD and to inform researchers, health care regulators, decision-makers and strategic governmental health data policy working groups on national and international levels about their availability and currently identified limitations. The objectives are as follows:

- to provide an initial overview of available Austrian healthcare RWD sources for research and decision-making purposes, data locations and data custodians,
- to test and improve the previously published checklist^13^,
- to discuss and conclude which data quality aspects should be applied to improve the use of RWD for scientific and regulatory purposes.

## Methods

To meet the objectives, we tapped into expert knowledge within and outside the group of authors, conducted interviews, and common desktop research using search engines and employing snowballing techniques, i.e., searching research articles on Austrian healthcare and extracting the RWD source used. We applied the following research strategies:

- Initially, based on a past survey^14^, we identified health data registers established by Austrian law.
- In addition, we searched the PubMed database for publications based on Austrian RWD sources (articles in the period from February 2017 to February 2022 including the criteria ((Austria[Affiliation]) AND (Austrian[Title/Abstract])) AND (data[Title/Abstract]).
- We then performed a targeted search for RWD on professional societies’ and universities’ websites.
- Finally, we searched international RWD directories (e.g., OrphaNet) for Austrian RWD.
- Fifth and finally, the authors of this paper used their practitioners’ knowledge to identify additional RWD sources in Austria.

Based on this search strategy, we extracted only healthcare-related RWD sources as described in the articles and listed those who fit the RWD definition as published previously^13^. We categorized results according to institutional data holder and category of the RWD source:

- For data holders, we differentiated between types of institutions that hold the data, including (1) expert communities (loose networks of experts without any formal organization), (2) professional societies (formally organized associations), (3) universities (organization under public law), (4) government institutions (ministries and public authorities including organization under direct state control based on private law), (5) hospitals, and (6) social insurance organizations.
- We categorized the RWD sources based on the collection’s main purpose derived from information available on the web and verified in interviews. “Main purpose” does not mean that the data cannot be used for other purposes; however, it was defined based on the intended use during RWD establishment (= database setup / inauguration). We identified seven main purposes: (1) clinical, (2) epidemiological, (3) quality assurance, (4) regulatory, (5) administrative, (6) research, and (7) informational.
- Finally, we categorized the subject of the RWD: (1) administrative data are data that are generated in administrative activities, (2) administrative registries also follow administrative purposes but have a legal basis, (3) biobanks store biological samples, (4) disease registries: the main data unit is a disease, (5) patient registries: the main data units are human subjects, (6) product registries: the main data units are products, (7) intervention registries: the main unit is an intervention, (8) health care databases include various health care data, and (9) observational studies.

**Figure 1:**
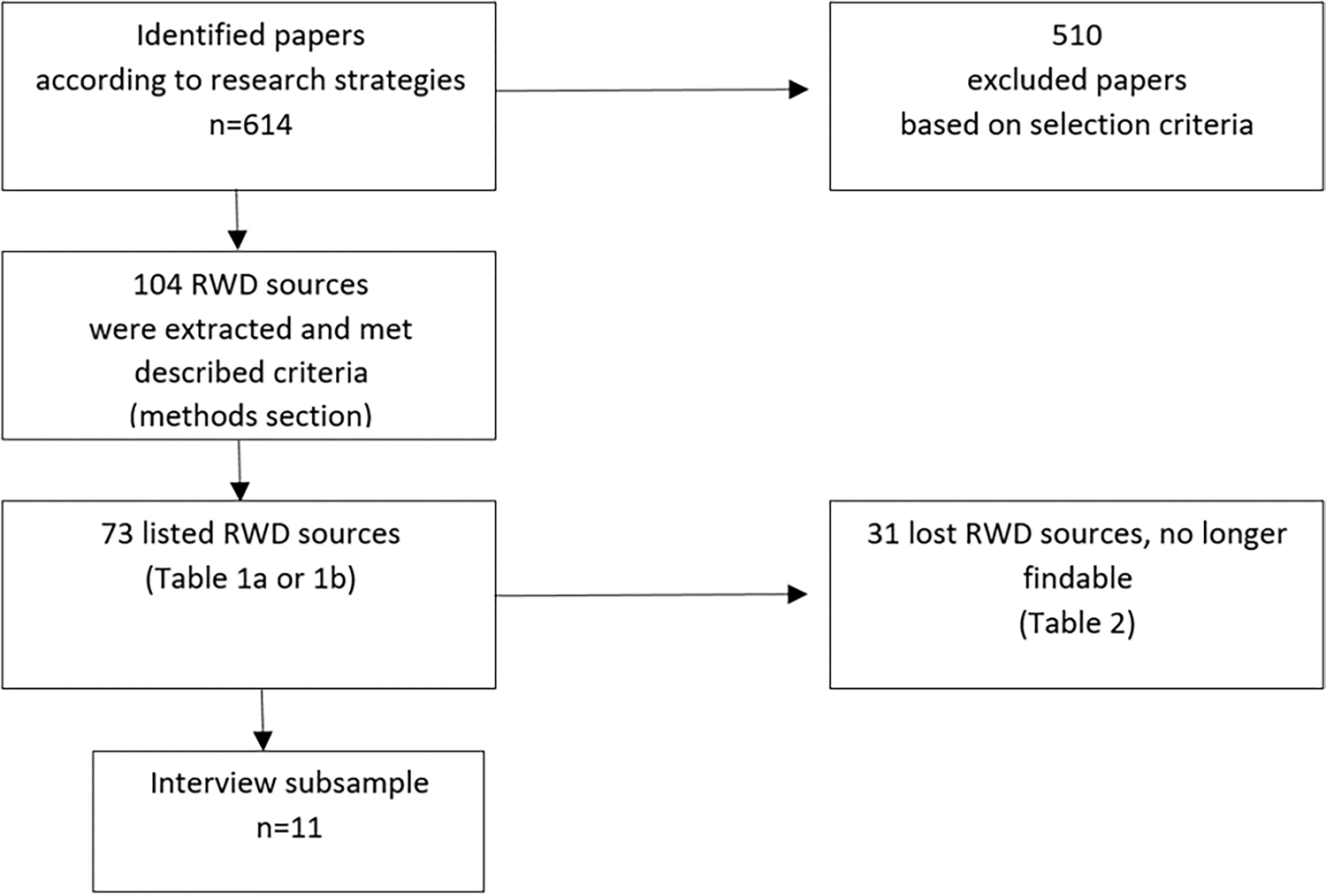
RWD source inclusion and selection process.

Following our objectives, we also conducted interviews with data holders on a subset of RWD sources out of the dataset “listed RWD sources” (Fig. 1). The sampling strategy was agreed upon by the author consortium and was used to create a representative RWD sample based on (1) purpose as well as (2) institutional type of data holder. During the interviews, we conducted a meticulous review of the checklist^13^ with the data holders, employing a systematic approach to ensure a comprehensive evaluation. To assess their own RWD sources, the interviewees utilized a rating scale for each quality criterion within the checklist, including options such as fully realized, partially realized, not realized, not realized but planned, and not applicable. Based on the participant information and consent form that we have completed with all the interview partners, we are able to utilize the aggregated and anonymized results in our work. The final scoring was determined by the authors.

## Results

We identified 73 out of 104 RWD sources that met the defined criteria and objectives (Supplementary Table A). Thirty-one out of 104 RWD sources mentioned in publications were no longer findable or accessible online (Supplementary Table B). Table 1 provides an overview what data holder group holds RWD sources in what category as outlined under methods.

**Table 1:** RWD main purpose and type of data holder matrix.

	Administrative	Clinical	Epidemiological	Quality assurance	Regulatory	Research	Total
Expert community		7	6	3		1	17
Government Organisation	6	2	7	6	6		27
Hospital (Association)		2	3	4			9
Other				1			1
Professional Society		1	5	4			10
Social Insurance Institution				1			1
University		3	3			2	8
Total	6	15	24	19	6	3	73

**Figure 2:**
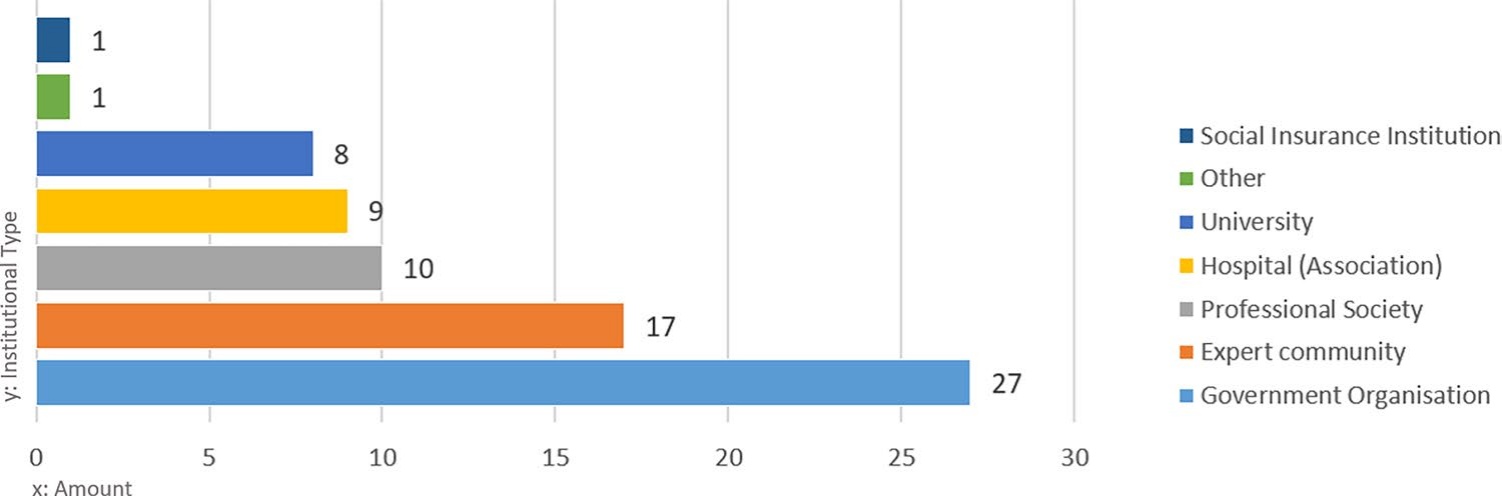
Amount of RWD sources per institutional type.

**Figure 3:**
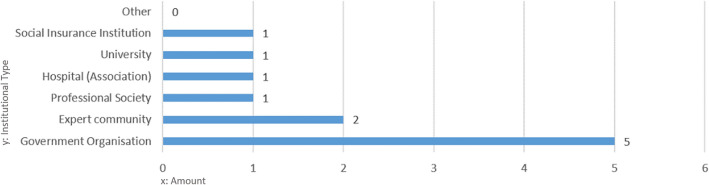
Distribution of institutional types of RWD holders among the interview sample.

We identified 30 different organisations holding and managing RWD sources (Supplementary Table C), which we further grouped into seven institutional types of RWD holders (Fig. 2). Expert communities and professional academic societies owned 27 verified RWD sources in Austria. All Austrian medical universities hold at least one RWD source. For the Austrian governmental organisations, all of the main institutions appeared as data holders (e.g., Federal Ministry of Social Affairs, Health, Care and Consumer Protection (BMSGPK), Federal Office for Safety in Health Care/Austrian Medicines and Medical Devices Agency (BASG/AGES) and Austrian National Public Health Institute Gesundheit Österreich GmbH (GÖG)), and this group holds 27 RWD sources. The Austrian social insurance is also amongst the RWD holders, which already shared specific data sets for research purposes. The selected interview sample reflects the overall distribution of institutional types of RWD holders, as shown in Fig. 3.

**Figure 4:**
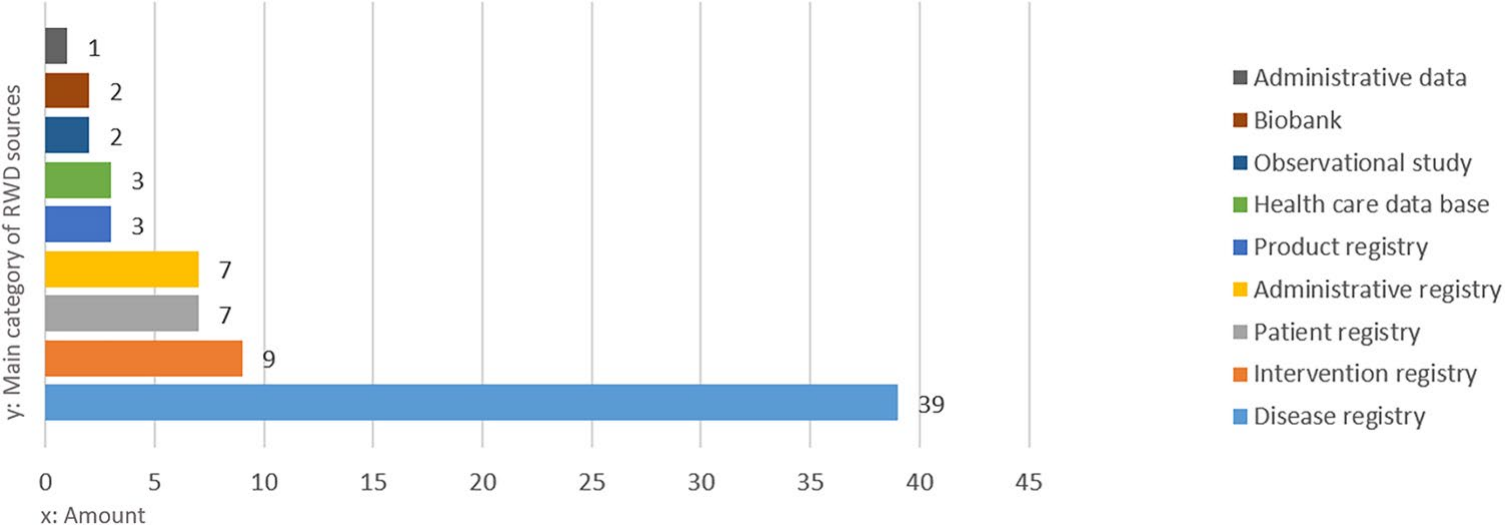
Main category of RWD sources.

**Figure 5:**
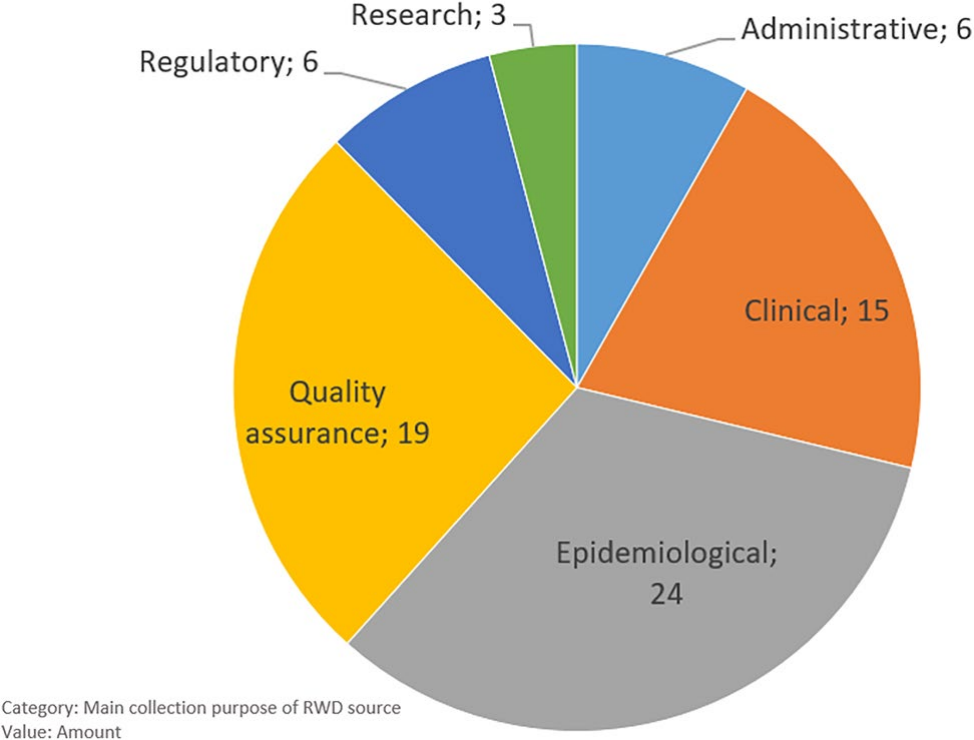
Distribution of the main collection purpose of RWD sources overall.

**Figure 6:**
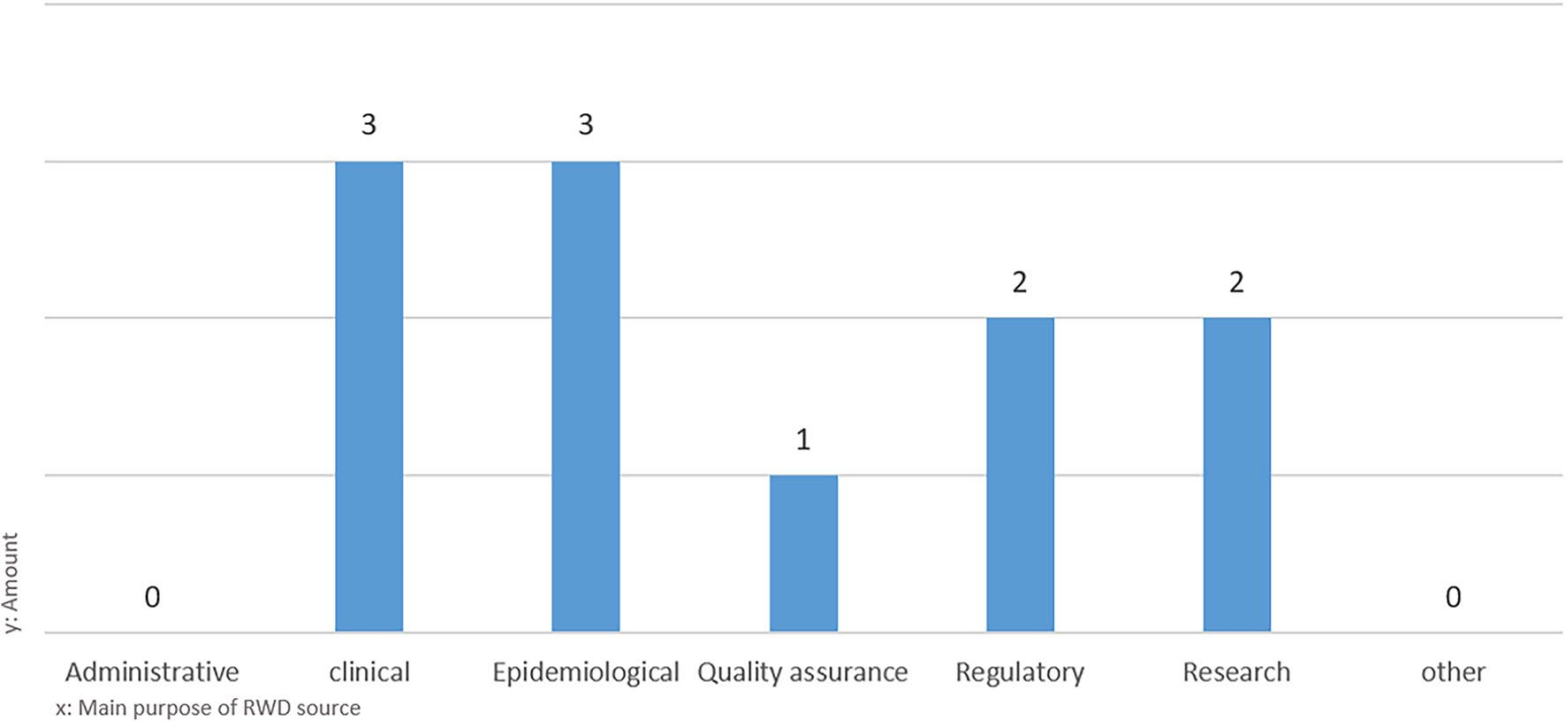
Distribution of the main purpose of the RWD source among the interview sample.

The majority of identified and verified RWD sources are registries (89%) followed by health care databases (4%), biobanks (3%), observational collections (3%) and administrative data collections (1%). Thirty-nine RWD sources belonged to the category “disease registry” (Fig. 4). The distribution of the main purpose mainly follows a functional differentiation: governmental organisations and social insurance carriers hold RWD sources with an administrative and quality assurance purpose. Governmental organisations are also central for RWD with an epidemiological and regulatory purpose (Fig. 5). Medical universities as well as professional organisations often run clinical RWD sources. More strikingly, there are only a few RWD sources whose main purpose lies in research (beyond clinical questions). Despite the small size of the subset consisting of 11 RWD sources, which were utilized for conducting interviews with the representative data holders, we present Fig. 6 as an overview to demonstrate that the distribution of the 'main purpose' among the subset is comparable to the dataset of '73 listed RWD sources' mentioned in Supplementary Table A.

**Figure 7:**
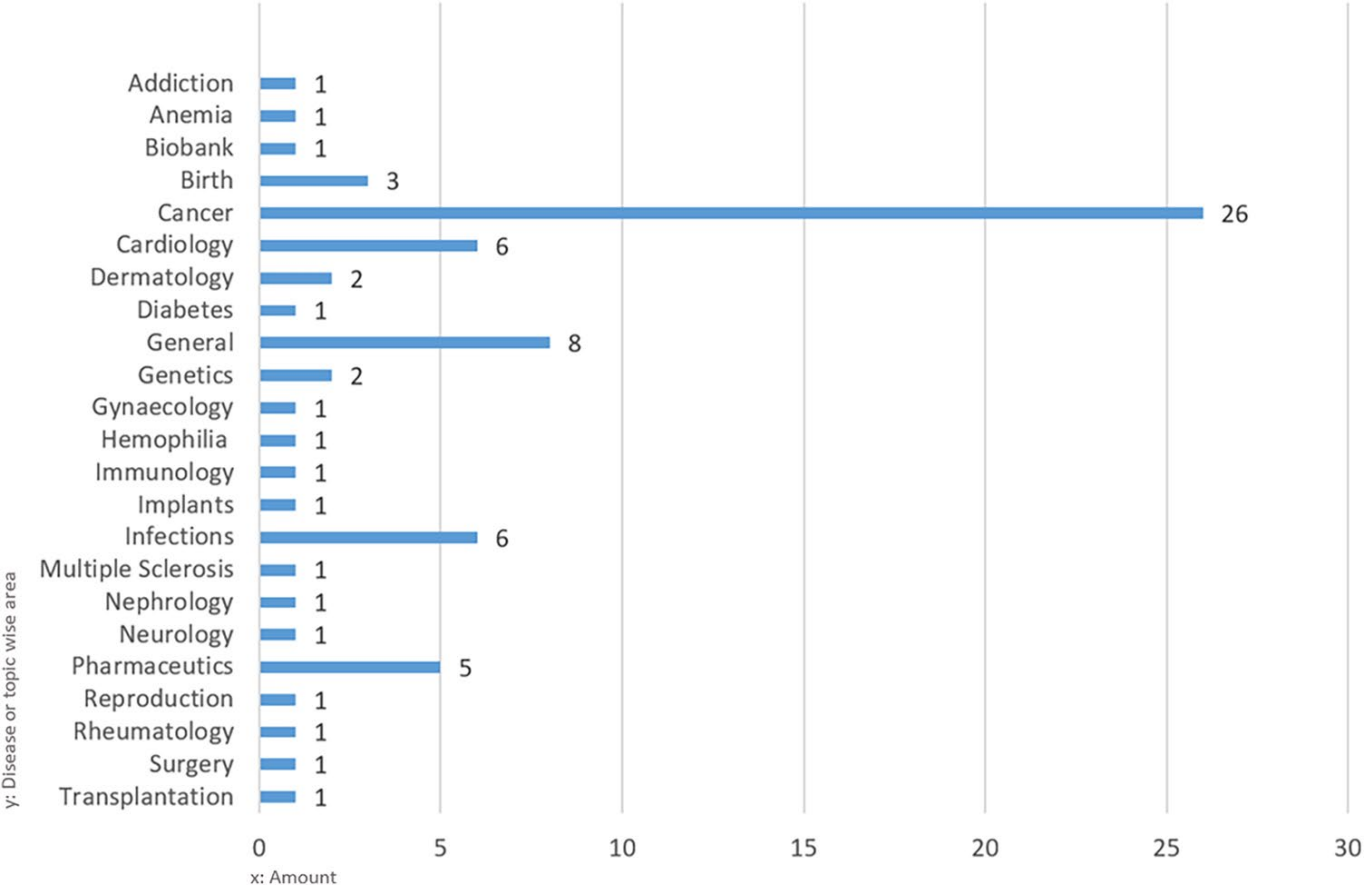
Disease or topic-wise areas of RWD sources in Austria.

Following our approach to cluster identified RWD sources by disease area or topic wise, most RWD sources in the clinical and/or epidemiological domain can be mapped to the disease area “cancer” (26 out of the 73, Fig. 7), as RWD sources in cardiovascular diseases do in quality assurance. Due to the strict regulation of the pharmaceutical domain, a high number of RWD for regulatory, administrative and quality assurance purposes exist. Only a few remaining RWD sources focus on other specific diseases.

**Figure 8:**
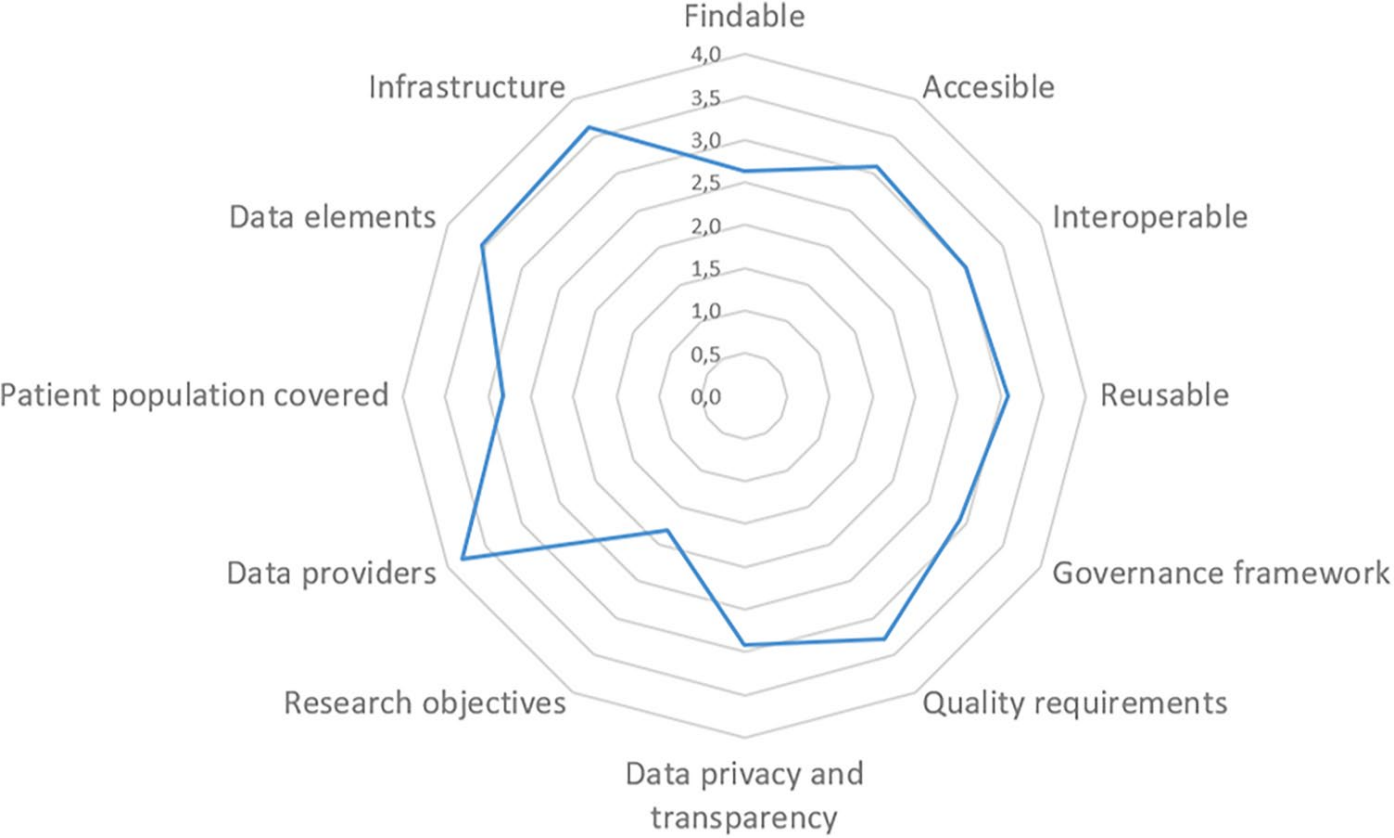
Achieved quality criteria of 11 examined RWD sources.

In line with our research objective to assess and enhance the previously published checklist^13^, the results of the conducted interviews, which were based on a subset of 11 RWD sources (as shown in Fig. 1), revealed that this particular subset of RWD sources already fulfilled numerous quality criteria outlined in the checklist. The parameters "Infrastructure”, "Data Elements", "Data Provider" and “Quality requirements” stood out as the most commonly fulfilled criteria (Fig. 8). Among the four FAIR Data Principles, the principle of 'Findable'—essentially referring to the ease of locating the data source through a website or online research—was found to be the least fulfilled when compared to the principles of 'Accessible', 'Interoperable', and 'Reusable'. This indicates that data owners should pay particular attention to addressing this fundamental principle. Based on our overall research approach and the experience we gained, the results of the interviews clearly demonstrate that the challenges we encountered during our own research, particularly in terms of "finding" the relevant RWD sources, were subjectively perceived as cumbersome and time-consuming. The quality criterion "data privacy and transparency" produced low ratings due to the ambiguous interpretation resulting from the type of regulations used, e.g., informed consent processes and GDPR for research vs. national regulations implemented by law. The same applied to the low rating of “Research objectives”, since RWD sources set up by law do not necessarily follow a research question or protocol such as the approach inherent to classic clinical research projects. This also concerned the parameter “Patient population covered” due to the heterogeneity and disease-specification not applying to the general population.

The interviews provided us with valuable feedback so that we were able to revise the checklist^13^ and interviewees had the opportunity to self-assess their own RWD sources utilizing the checklist. The overall average results of these self-assessments from the 11 interviews are presented in Fig. 8. The checklist has undergone minor revisions, including the addition of references and improvements in language. We have added headlines and an additional column with for rating options. However, the sub-element "core RWD set collected for RWD use case or purpose" in the data-elements section has been removed for usability reasons. The revised version of the checklist can be found in Table 2.

**Table 2:** Checklist on quality criteria for RWD revised version 2.0.

Main criterion	Sub-criterion	Rating options for (self)assessment
Data management and stewardship	The "FAIR Data Principles" formulate principles that sustainable, reusable research data and research data infrastructures must meet Definitions see here: https://www.go-fair.org/fair-principles/	Fully realized Partially realized Not realized Not realized but planned Not applicable
Governance framework	Available policy for collaborations with external organizations Governance structure for decision-making on requests for collaboration Available templates for research/data-sharing contracts Involvement of Patient Organizations	Fully realized Partially realized Not realized Not realized but planned Not applicable
Quality requirements	High RWD quality standards are implemented—such as: completeness—accuracy—timeliness—comparability Process in place for ongoing data quality assessments Processes in place for quality planning, control, assurance and improvement Data verification (method and frequency of verification) Auditing practice	Fully realized Partially realized Not realized Not realized but planned Not applicable
Data privacy & transparency	Informed consent form and its validity for research purposes according to GDPR, EHDS and relevant national regulations	Fully realized Partially realized Not realized Not realized but planned Not applicable
Research objectives	*Note—Only applicable if the primary purpose of the RWD is research* Well defined research question outlined in a research plan Available documentation, protocol or proposal which describes purpose of RWD use and rational that the RWD data sources adequately addresses the research questions (e.g. study protocol) Approval of RWD use of independent review board/ethics committee Protocol should follow the Declaration of Helsinki and furthermore the Declaration of Taipei [26] on Research on Health Databases, Big Data and Biobanks should be taken into account	Fully realized Partially realized Not realized Not realized but planned Not applicable
Data providers	Description of data providers, such as patients, carers or health care professionals, their geographical area and any selection process (inclusion and exclusion criteria) that may be applied for their acceptance as data providers	Fully realized Partially realized Not realized Not realized but planned Not applicable
Patient population covered	Description of the type of patient population (disease, condition, time period covered, procedure), which defines the criteria for patient eligibility Relevance of setting and catchment area Clarity on patients’ inclusion and exclusion criteria Methods applied to minimise selection bias and loss to follow-up	Fully realized Partially realized Not realized Not realized but planned Not applicable
Data elements	Definition, dictionary and format of data elements Standards and terminologies applied Capabilities and plans for amendments of data elements	Fully realized Partially realized Not realized Not realized but planned Not applicable
Infrastructure	High quality systems for RWD collection, recording and reporting, including timelines Capability (and experience) for expedited reporting and evaluation of severe suspected adverse reactions in RWD collection Capability (and experience) for periodic reporting of clinical outcomes—ideally patient reported outcomes—and adverse events reported by physicians, at individual-patient level and aggregated data level Capability (and experience) for data cleaning, extraction, transformation and analysis Capability (and experience) for data transfer to external organisations Capabilities for amendment of safety reporting processes	Fully realized Partially realized Not realized Not realized but planned Not applicable

## Discussion

Our research approach to identify RWD sources out of publications reveals various challenges concerning the availability and accessibility of the national RWD landscape. The considerable effort invested in this work to identify RWD resources underscored the importance of providing a central directory for RWD sources aligned with DGA^7^ and EHDS^15^ requirements (e.g., data catalogues) to facilitate research with high-quality data sets, which could serve as a valuable resource for all stakeholders. The time and resources required to search for and locate each of the identified RWD sources were a major obstacle to utilizing the available data sets in a more efficient manner.

Several RWD sources identified in the search process were not findable online (31 of 104 RWD sources, Supplementary Table B). It remains unclear if adequate metadata descriptions of these RWD sources were just unavailable or if they have been deleted since. This, however, puts the research integrity of these sources, notably data transparency and reproducibility, into question. This highlights the importance of data holders ensuring the long-term accessibility of collected RWD, enabling their reuse for (secondary) research purposes. Without such accessibility features, the potential benefits of using RWD for research, public health policy, and society in general cannot be reached.

RWD with a dedicated research purpose used in the analysed articles were rather a national exception. Predominantly, publications on RWD data sets are characterised by the secondary use of quality assurance data or epidemiological RWD, indicating a gap in the integration of academic research into public health policy-making in Austria. This suggests that aside the primary intention to establish a register, the possibility of opening the register data for further research or decision-making purposes (secondary data use) was not or only partially considered. The limited availability of RWD collected for research purposes hinders the potential to develop evidence-based policies and strategies that could positively impact public health outcomes in the country.

Expert communities and professional societies hold a substantial number of RWD sources. However, these organizations are often characterized by lacking adequate resources to maintain robust data management practices, e.g. up-to-date content and long-term availability. Due to missing directories, lacking online meta data descriptions and undefined rules for third party access, these RWD sources appear to be data silos or “club good” for “insiders” and cannot provide any benefit for healthcare research or policymaking.

The population of RWD data holders in Austria is quite diverse ranging from small professional societies to large public authorities. While this diversity could prove beneficial, this is also a source of the siloization of health data in Austria as demonstrated by the fact that barely any article in our sample used more than one data set in each publication due to legal and technical restrictions.

These findings prompt a critical discussion regarding the current state of working with or setting up RWD sources that do not adhere to FAIR data principles. It raises the question of whether such practices can still be considered state-of-the-art demonstrating a striking contrast to the initiatives on the European level as stated in the introduction. A substantial share of the RWD sources was not findable (Table 2). Accessibility was another major issue, either based on the lacking “findability”, or if findable on undefined rules for third party access. This concerns also public RWD where some institutions could use administrative datasets based on contracts, but given the transaction costs, this impedes smaller research groups and individual researchers to use these data. Therefore, the prevalence of data silos and the lack of data interoperability and standardization^12^ continues to pose challenges in this fragmented RWD landscape impeding the potential of RWD in general. The shortcomings of the RWD landscape in Austria have shown that the previously published RWD quality checklist^13^ and the feedback from the interviewees were valuable resources to inform future RWD efforts to consider multifunctional use of the data in the long term. A response was: "We would have needed this checklist before we built the registry".

Furthermore, the findings of the interviews confirmed our initial assumption that research readiness for secondary purposes and broader applicability were albeit often, forgotten during the inauguration of RWD sources. In the assessment of the checklist by the interviewees, registers/cohorts dedicated to specific purposes tended to receive high scores in terms of research readiness. However, their usefulness was limited due to the prevailing data siloization. This lack of data integration and interoperability prevents researchers from harnessing the full benefits of these "research-ready" datasets, leading to their underutilization. Interestingly, some of the most comprehensive and interesting RWD sources out of the subset score low on the checklist criteria, putting their value as RWD source into question. However, following our broad definition of RWD^13^ not every RWD source is inaugurated based on a research objective (e.g. health care claims data). This might highlight the prevailing marginal status of RWD utilization, as these valuable datasets remain underutilized and underappreciated in the research community. We also received valuable and constructive suggestions on how to further improve or adapt the criteria listed in the checklist so that it can be used more broadly (Table 2).

## Conclusion

The health data landscape changes constantly due to new data collection points, cheaper and faster availability of omics data, digital health and digital care pathways, imaging technology and artificial intelligence. This evolution creates opportunities not only for healthcare research and development but also for public health and health policy^6^. This necessitates increased coordination, the creation of common (meta)data standards and interoperability to avoid siloization and to maximise the benefits of RWD through data exploration in linked data sets, which are able to represent the complexities of public and individual health issues.

However, the legislative environment is not yet ready to support RWD within the boundaries of fundamental rights. This is for several reasons, not all of them being purely of a legal nature. Strictly legally speaking, Austria already made a major attempt to increase access to secondary use of data via several reforms of the federal law on the organisation of research (“Forschungsorganisationsgesetz”) and of the law on statistics (“Bundesstatistikgesetz”) in 2018^16^ and in 2021^17,18^, respectively. The aim of these reforms was to increase the accessibility of existing (personal) data for research purposes. However, for several reasons, including the lack of secondary legislation on a ministerial level that would have been needed and due to legal complexity, these attempts have not yet sufficiently reached their goals. The already complex national situation faces new challenges by the planned European legislative initiatives, in particular the DGA^7^ and the EHDS Act^15^. The DGA aims to improve data sharing and data reuse within the European Union (EU) by introducing, inter alia, competent bodies (Art. 7), single information points (Art. 8), data intermediation services (Art. 10) and public registers of recognised data altruism organisations (Art. 17). The EHDS will likely introduce a whole chapter on the secondary use of electronic health data (Chapter IV), introducing health data access bodies (Art. 36), rules on data altruism in health (Art. 40), a cross-border infrastructure for secondary use of electronic health data (HealthData@EU) (Art. 52) and new governance bodies such as the EHDS Board (Art. 64). Whereas these European attempts have the potential to improve the accessibility of RWD, there exists at the same time a significant risk of even more legal complexity by legal inconsistency, national deviations and unclarity as an unwanted offspring of these initiatives.

High-quality criteria for RWD are key for improved data utilization in research and healthcare decision-making^4^. The herein provided improved checklist (Table 2) may also support authorities and government institutions in their attempt to ensure data quality for the whole sector, in particular with regard to the implementation of the DGA and the coming EHDS as well as national and European activities of open science. RWD sources can foster a more open culture of data sharing and reuse, which is unfortunately almost absent in the currently reviewed health data sector.

We also call for a critical, scientifically driven analysis of the regulatory environment, together with an attempt to simplify the legal landscape, and more ambitious and structured governance activities regarding health data, in particular for a more comprehensive approach to data collection, considering the potential for future research and wider utilization. Multipurpose datasets may increase efficiency and may act as a boost for research on topics that are often neglected due to the lack of data. A significant improvement in data utilization could be achieved through better linking of data from both public and private sources. Our findings emphasize the creation of a comprehensive data strategy in the healthcare domain, especially in the reviewed national framework in Austria.

On the upside, Austria employs already sector-specific personal identifiers to link data across data sets without compromising privacy and data protection (the so-called "bereichsspezifische Personenkennzeichen (bPK)"), and the recently established Austria Microdata Center (AMDC) at Statistics Austria can serve as a role model for the use of administrative and statistical data for research (legally, technically, organisational).

Future legislative developments at the EU level (e.g. EHDS^15^ or pharma legislation^9^), the efforts of the HMA/EMA Big Data Steering Group^19^ and in particular the European Medicines Regulatory Network (EMRN) and the RWD for Decision Making Network (RWD4DM) will provide significant impetus.

Recent national developments such as the government’s introduction of the Digital Austria Act^20^ in mid-2023 and the recommendations of the “Digitalization and Registries Working Group” to create an Austrian health data space^21^ indicate that there is more awareness of better data use in national health policy. Further encouraging signals regarding the improvement of the secondary use of health data can be found in the “eHealth Strategy”^22^ as well as in the national healthcare measures within the federal finance act^23^ presented in November 2023.

While governments have a responsibility to create clear legal frameworks, data holders have no less responsibility to ensure that RWD is made accessible and usable in accordance with new regulations. However, if the goals and plans set are not followed by action, then no added value can be generated from the use of RWD for each individual, society and the healthcare system. In conclusion, the findings underscore the need for:

- a central directory of RWD that also helps to enact quality standards on data sets,
- raising awareness and compliance with data standards, in particular the “Findable”–“Accessible”–“Interoperable”–“Reusable” (FAIR) data principles given that a substantial share of RWD is neither findable nor accessible,
- a more strategic approach to think about the roles and features of existing and future data sets, in particular by including the research purpose in RWD,
- resolving issues to warrant sustainable data management by providing adequate resources,
- a fundamental legal work and willingness to simplify the existing national legislation as well as to adapt it in an RWD-supportive manner to the (reformed) EU-layer of relevant secondary law and to,
- leave data silo-ization behind and start creating interoperable data sets.

### Supplementary Information

Supplementary Tables.

## Data Availability

The datasets generated during the PubMed research approach described in the methods section are available from the corresponding author upon reasonable request. All data analysed during this study are included in this published article (and its Supplementary Information files).
