# Supplementary material for: A national evaluation analysis and expert interview study of real-world data sources for research and healthcare decision-making
Source: Sci Rep. 2024 Apr 28;14:9751. doi: 10.1038/s41598-024-59475-9 (PMC11056370; doi:10.1038/s41598-024-59475-9)
Supplement: Supplementary file 1 — Supplementary Tables. [file 41598_2024_59475_MOESM1_ESM.docx]

**A national evaluation analysis and expert interview study of real-world data sources for research and healthcare decision-making**

# Supplementary Information – Tables

Legends

Supplementary table A. List of verified RWD sources in Austria.

Supplementary table B. List of unverifiable lost RWD sources.

Supplementary table C. Identified RWD holder.

Supplementary Table D. Identified RWD holder.

Supplementary Table E. Checklist on Quality criteria for RWD revised version 2.0.

## Supplementary Table A. List of verified RWD Sources in Austria.

| **ID** | **Name of RWD Source** | **Area** | **Data holder** | **Type Data Holder** | **RWD Category 1** | **Purpose type 1** | **Original Online Source** | **Use Case / Publication** |
| --- | --- | --- | --- | --- | --- | --- | --- | --- |
| 1 | AGMT HMA Registry | Cancer | Arbeitsgemeinschaft medikamentöse Tumortherapie (AGMT) | Expert community | disease registry | clinical | https://www.agmt.at/hma-registry/ | https://doi.org/10.1016/j.leukres.2017.05.006 |
| 2 | Austrian Hospital Discharge Register (AHDR) - Spitalsentlassungsstatistik | General | Statistik Austria | Government Organisation | disease registry | epidemi- ological | <https://www.statistik.at/statistiken/bevoelkerung-und-soziales/gesundheit/gesundheitsversorgung-und-ausgaben/gesundheitsversorgung-stationaer-spitalsentlassungen> | <https://link.springer.com/article/10.1007/s00198-021-06086-z> |
| 3 | Austrian Haemophilia Registry | Hemophilia | Österreichische Hämophilie Gesellschaft | Professional Society | disease registry | quality assurance | <https://bluter.at/wp/osterr-hamophilie-register/> | <https://www.thieme-connect.de/products/ejournals/abstract/10.1055/s-0038-1675354> |
| 4 | Austrian GIST Registry (Gastrointestinal Stromatumors) | Cancer | Österreichische Gesellschaft für Chirurgie - OEGCH | Professional Society | disease registry | epidemi- ological | <https://oegch.at/qualitaetssicherung/gist-register/> |  |
| 5 | Austrian Brain Tumor Registry | Cancer | Medizinische Universität Wien | University | disease registry | epidemi- ological | <https://www.meduniwien.ac.at/hp/npc/allgemeine-informationen/aufgaben/das-oesterreichische-hirntumorregister/#:~:text=Das%20am%20KIN%20verortete%20%C3%96sterreichische,dem%20%C3%96sterreichische%20Krebsregister%20innerhalb%20der> | <https://doi.org/10.1007/s11060-009-9938-9> |
| 6 | MS Register | Multiple Sclerosis | Österreichische Gesellschaft für Neurologie | Professional Society | intervention registry | epidemi- ological | <https://www.oegn.at/ms-register-der-oegn/> | https://doi.org/10.1007/s00415-021-10559-w |
| 7 | Austrian Myeloma Registry | Cancer | Österreichische Gesellschaft für Hämatologie & Medizinische Onkologie | Professional Society | disease registry | quality assurance | https://www.oegho.at/forschung/klinische-register/ | <https://doi.org/10.1371/journal.pone.0147381> |
| 8 | Kooperatives Photodermatosen Register | Derma- tology | Medizinische Universität Graz | University | disease registry | clinical | <https://www.medunigraz.at/universitaetskliniken/dermatologie-und-venerologie/forschung/bioimmuntherapie/register-und-datenbanken> |  |
| 9 | Biobanking and BioMolecular resources Research Infrastructure Austria | Biobank | BBMRI.at | other | biobank | quality assurance | <https://bbmri.at/> | <https://doi.org/10.1515/cclm-2019-0491> |
| 10 | Hautkrebssyndrom-Register Graz | Cancer | Medizinische Universität Graz | University | disease registry | clinical | <https://www.medunigraz.at/universitaetskliniken/dermatologie-und-venerologie/forschung/bioimmuntherapie/register-und-datenbanken> |  |
| 11 | AMIG Mesotheliom Datenbank | Cancer | Austrian Mesothelioma Interest Group | Expert community | disease registry | epidemi- ological | <http://amig.at/was-wir-tun/amig-mpm-datenbank> | <https://doi.org/10.1007/s00508-016-1037-2> |
| 12 | Austrian National Cardiac Catheterization Laboratory Registry (ANCALAR) | Cardiology | Österreichischen Kardiologischen Gesellschaft | Professional Society | intervention registry | quality assurance | <https://iik.i-med.ac.at/index.php?param=register> | <https://link.springer.com/article/10.1007/s00508-019-01599-4> |
| 13 | PsoRA Psoriasis Registry Austria | Derma- tology | Medizinische Universität Graz | University | disease registry | research | <https://psora.medunigraz.at/> | <https://onlinelibrary.wiley.com/doi/10.1111/bjd.19701> PMID: 28898541 DOI: 10.1111/jdv.14583 |
| 14 | Austrian Sacrocolpopexy Registry | Gynae-cology | Österreichische Arbeitsgemeinschaft für Urogynäkologie & Rekonstruktive Beckenbodenchirurgie | Expert community | intervention registry | quality assurance | <https://www.urogyn.at/studie/sakrokolpopexie-register> | <https://www.jmig.org/article/S1553-4650(20)31109-2/fulltext> |
| 15 | Central Data Registry of The European Competence Network on Mastocytosis | Immun-ology | Medizinische Universität Wien | University | disease registry | epidemi- ological | <https://ecnm.meduniwien.ac.at/registry/> | <https://doi.org/10.1016/j.jaip.2023.02.021> |
| 16 | Austrian Stroke Unit Registry | Cardiology | Gesundheit Österreich GmbH | Government Organisation | disease registry | quality assurance | <https://goeg.at/Qualitaetsregister> | <https://journals.plos.org/plosone/article?id=10.1371/journal.pone.0214980> |
| 17 | Austrian substitution registry - Suchtmittelregister und Substitutionsregister | Addiction | Bundesministerium für Soziales, Gesundheit, Pflege und Konsumentenschutz | Government Organisation | intervention registry | regulatory | <https://www.oesterreich.gv.at/themen/gesundheit_und_notfaelle/sucht/2/1/Seite.1520650.html> | <https://harmreductionjournal.biomedcentral.com/articles/10.1186/s12954-021-00473-9> |
| 18 | Austrian Toxoplasmosis Register | Infections | Medizinische Universität Wien | University | disease registry | epidemi- ological | <https://www.meduniwien.ac.at/web/ueber-uns/news/detail/toxoplasmose-screening-verringert-infektionsrate/> | <https://journals.plos.org/plosntds/article?id=10.1371/journal.pntd.0005648> |
| 19 | Diabetesregister Tirol | Diabetes | LIV - Landesinstitut für Integrierte Versorgung Tirol | Hospital (Association) | disease registry | quality assurance | <https://www.iet.at/page.cfm?vpath=register/diabetesregister> | <https://www.iet.at/data.cfm?vpath=publikationen210/drt/drt-bericht-2021> |
| 20 | Klinisches Tumorregister Österreich für gynäkologische Tumoren | Cancer | LIV - Landesinstitut für Integrierte Versorgung Tirol | Hospital (Association) | disease registry | clinical | <https://www.iet.at/page.cfm?vpath=register/qualitaetssicherung-ago> | <https://doi.org/10.1007/s00508-023-02162-y> |
| 21 | Epidemiologisches Tumorregister Tirol | Cancer | LIV - Landesinstitut für Integrierte Versorgung Tirol | Hospital (Association) | disease registry | epidemi- ological | <https://www.iet.at/page.cfm?vpath=register/tumorregister> | <https://doi.org/10.1007/s00404-021-06117-4> |
| 22 | Österreichischea Nationales Krebsregister | Cancer | Statistik Austria | Government Organisation | disease registry | epidemi- ological | <https://www.statistik.at/statistiken/bevoelkerung-und-soziales/gesundheit/krebserkrankungen> | <https://www.statistik.at/services/tools/services/publikationen/detail/1411> |
| 23 | Außerklinisches Geburtenregister Österreich | Birth | LIV - Landesinstitut für Integrierte Versorgung Tirol | Hospital (Association) | patient registry | quality assurance | <https://www.liv.tirol/page.cfm?vpath=datenmanagement-und-analytik/epidemiologie> |  |
| 24 | Geburtenregister Österreich | Birth | LIV - Landesinstitut für Integrierte Versorgung Tirol | Hospital (Association) | patient registry | quality assurance | <https://www.liv.tirol/page.cfm?vpath=datenmanagement-und-analytik/epidemiologie> | https://doi.org/10.1055/a-1911-1996 |
| 25 | Tumordatenbank für Tumorboard Oberösterreich | Cancer | Tumorzentrum Oberösterreich | Expert community | disease registry | quality assurance | <https://www.tumorzentrum.at/ueber-uns/kennzahlen> |  |
| 26 | Kärntner Tumorregister | Cancer | Klinikum Klagenfurt | Hospital (Association) | disease registry | epidemi- ological | <https://www.gesundheitsfonds.at/aufgaben1/qualitaetssicherung/tumorboard-tumorregister> | https://www.ktn.gv.at/DE/repos/files/ktn.gv.at/Abteilungen/Abt5/Dateien/TRK%20Bericht%202013.pdf?exp=435462&fps=7fe3d721374b383d1d872cc19689660ff0e791af |
| 27 | NÖ Onkologie-Informations-System (OIS) | Cancer | Niederösterreichischen Landeskliniken-Holding | Hospital (Association) | disease registry | clinical | <https://www.cgm.com/aut_de/magazin/artikel/2021/november-1/das-noe-onkologie-informationssystem.html> | <https://doi.org/10.1007/s43831-021-0036-9> |
| 28 | Tumorregister Salzburg | Cancer | Uniklinikum Salzburg | Hospital (Association) | disease registry | epidemi- ological | <https://salk.at/1136.html> |  |
| 29 | Tumorregister Vorarlberg | Cancer | Arbeitskreis für Vorsorge- und Sozialmedizin | Expert community | disease registry | epidemi- ological | <https://www.aks.or.at/science/cancer-registry/> | https://doi.org/10.1186/s12885-017-3683-9 |
| 30 | Arzneimittelspezialitätenregister | Pharma-ceutics | Bundesamt für Sicherheit im Gesundheitswesen | Government Organisation | product registry | regulatory | <https://www.basg.gv.at/fuer-unternehmen/zulassung-life-cycle/faq-zulassung-life-cycle/arzneispezialitaetenregister> | https://aspregister.basg.gv.at/aspregister/faces/aspregister.jspx |
| 31 | Stroke Card | Cardiology | Medizinische Universität Innsbruck | University | disease registry | clinical | <https://www.thieme-connect.de/products/ejournals/html/10.1055/a-1101-8949> |  |
| 32 | Austrian performance-based hospital financing system (Leistungsorientierten Krankenanstaltenfinanzierung) | General | Bundesministerium für Soziales, Gesundheit, Pflege und Konsumentenschutz | Government Organisation | administrative data | adminis-trative | <https://www.sozialministerium.at/Themen/Gesundheit/Gesundheitssystem/Krankenanstalten/Leistungsorientierte-Krankenanstaltenfinanzierung-(LKF).html> | https://doi.org/10.1007/s00063-017-0391-9 |
| 33 | Social Health Insurance Data from LeiCon-Database (Leistungscontrolling Datenbank) | General | Österreichische Sozialversicherung | Social Insurance Institution | health care data base | quality assurance | <https://www.sozialversicherung.at/cdscontent/?contentid=10007.849899&portal=svportal> | 10.1111/ecc.13423 10.1016/j.diabres.2021.108758 10.1007/s00784-019-03090-w 10.3390/ijerph18136891 10.1186/s10194-018-0864-0 10.1038/s41467-019-10914-6 10.1111/crj.12968 10.1007/s00223-019-00611-3 10.3390/jcm9113398 10.1159/000479696 10.1136/annrheumdis-2019-215714 10.1016/j.ijcard.2017.02.096 10.1002/trc2.12014 10.1002/gps.5506 10.1007/s10198-021-01298-w |
| 34 | AGMT Austrian CLL (chronic lymphocytic leukemia) Registry | Cancer | Arbeitsgemeinschaft medikamentöse Tumortherapie (AGMT) | Expert community | disease registry | clinical | <https://www.agmt.at/agmt_cll-reg/> |  |
| 35 | AGMT Austrian Myeliod Registry (aMYELOIDr) | Cancer | Arbeitsgemeinschaft medikamentöse Tumortherapie (AGMT) | Expert community | disease registry | clinical | https://www.agmt.at/myeloid-registry/ | 10.1111/ecc.13154 |
| 36 | AGMT Autoimmune Hemolytic Anemia (AIHA) Registry with corresponding Biobank | Anemia | Arbeitsgemeinschaft medikamentöse Tumortherapie (AGMT) | Expert community | disease registry | epidemi- ological | <https://www.agmt.at/aiha-registry/> |  |
| 37 | AGMT Covid-19 Registry | Infections | Arbeitsgemeinschaft medikamentöse Tumortherapie (AGMT) | Expert community | disease registry | clinical | <https://www.agmt.at/register-covid-19/> | https://doi.org/10.1007/s11357-021-00352-y |
| 38 | AGMT Lung Cancer Registry | Cancer | Arbeitsgemeinschaft medikamentöse Tumortherapie (AGMT) | Expert community | disease registry | epidemi- ological | https://www.agmt.at/register-lungca/ |  |
| 39 | AGMT Metastatic Breast Cancer (MBC) Registry | Cancer | Arbeitsgemeinschaft medikamentöse Tumortherapie (AGMT) | Expert community | disease registry | epidemi- ological | <https://www.agmt.at/mbc-registry/> | https://doi.org/10.1186/s13058-021-01492-x |
| 40 | AGMT PTCL Registry | Cancer | Arbeitsgemeinschaft medikamentöse Tumortherapie (AGMT) | Expert community | disease registry | epidemi- ological | <https://www.agmt.at/ptcl-registry/> |  |
| 41 | AGMT ALL Registry | Cancer | Arbeitsgemeinschaft medikamentöse Tumortherapie (AGMT) | Expert community | disease registry | clinical | <https://www.agmt.at/all-registry/> |  |
| 42 | AGMT BV-NIS Austrian Brentuximab Vedotin observational study | Pharma-ceutics | Arbeitsgemeinschaft medikamentöse Tumortherapie (AGMT) | Expert community | observational study | clinical | <https://www.agmt.at/bv-nis/> |  |
| 43 | AGMT NGS Registry | Cancer | Arbeitsgemeinschaft medikamentöse Tumortherapie (AGMT) | Expert community | disease registry | quality assurance | <https://www.agmt.at/ngs-registry/> | https://doi.org/10.1016/j.esmoop.2021.100233 |
| 44 | Colorectal Cancer Study of Austria (CORSA) | Cancer | Medizinische Universität Wien | University | observational study | research | <https://krebsforschung.meduniwien.ac.at/forschung/forschungsschwerpunkte/zellulaere-und-molekulare-tumorbiologie/andrea-gsur/research-projects/corsa/> | 10.1016/j.cgh.2021.04.023 |
| 45 | ELGA e-Befunde | General | ELGA GmbH | Government Organisation | health care data base | clinical | <https://www.elga.gv.at/> | 10.1111/ddg.14511 |
| 46 | Genanalyse-Register | Genetics | Bundesministerium für Soziales, Gesundheit, Pflege und Konsumentenschutz | Government Organisation | administrative registry | adminis-trative | <https://www.verbrauchergesundheit.gv.at/gentechnik/rechtoe/GTG/Genregister.html> |  |
| 47 | Zentrale Liste Ringversuche | Genetics | Bundesministerium für Soziales, Gesundheit, Pflege und Konsumentenschutz | Government Organisation | administrative registry | regulatory | <https://www.verbrauchergesundheit.gv.at/gentechnik/humanm/GTG_Register.html> |  |
| 48 | Liste der Meldungen zu Vertriebseinschränkungen von Arzneispezialitäten | Pharma-ceutics | Bundesamt für Sicherheit im Gesundheitswesen | Government Organisation | administrative registry | adminis-trative | <https://medicineshortage.basg.gv.at/vertriebseinschraenkungen/faces/adf.task-flow?_id=main-btf&_document=WEB-INF/main-btf.xml> |  |
| 49 | Liste gemäß Verordnung über die Sicherstellung der Arzneimittelversorgung | Pharma-ceutics | Bundesamt für Sicherheit im Gesundheitswesen | Government Organisation | product registry | regulatory | https://www.basg.gv.at/marktbeobachtung/amtliche-nachrichten/detail/verordnung-zur-sicherstellung-der-arzneimittelversorgung-tritt-mit-1-april-2020-in-kraft#:~:text=Die%20%E2%80%9EListe%20gem%C3%A4%C3%9F%20Verordnung%20%C3%BCber,BASG%20ein%20Parallelexportverbot%20erhalten%20haben. |  |
| 50 | Österreichische Register für Medizinprodukte | General | Gesundheit Österreich GmbH | Government Organisation | administrative registry | quality assurance | <https://medizinprodukteregister.at/> |  |
| 51 | Register zur Qualitätssicherung in der Herzchirurgie | Cardiology | Gesundheit Österreich GmbH | Government Organisation | intervention registry | quality assurance | https://goeg.at/Koordinationsstelle_Qualitaetsregister |  |
| 52 | Herzschrittmacher-, ICD-, Looprecorder-Register | Cardiology | Gesundheit Österreich GmbH | Government Organisation | administrative registry | regulatory | https://goeg.at/Koordinationsstelle_Qualitaetsregister |  |
| 53 | Register Nicht-Interventionelle Studien (NIS) | General | Bundesamt für Sicherheit im Gesundheitswesen | Government Organisation | administrative registry | regulatory | <https://www.basg.gv.at/gesundheitsberufe/klinische-studien/nicht-interventionelle-studien-nis> |  |
| 54 | Pharma-Preisinformationssystem (PPI) | General | Gesundheit Österreich GmbH | Government Organisation | administrative registry | adminis-trative | <https://goeg.at/Pharma-Preisinformationssystem> |  |
| 55 | BIOREG Register für Biologica, Biosimilars und tsDMARDs bei der Behandlung von entzündlichen rheumatischen Erkrankungen | Rheuma-tology | Trägerverein für das Österreichische Register für Biologica, Biosimilars und tsDMARDs bei der Behandlung von entzündlichen rheumatischen Erkrankungen (BioReg) | Expert community | disease registry | clinical | https://www.bioreg.at/stats/ |  |
| 56 | Österreichisches Parkisnonregister ÖPAR | Neurology | Österreichische Parkinsongesellschaft | Professional Society | disease registry | clinical | <https://www.parkinson.at/medizin-forschung/projekte-publikationen.html> |  |
| 57 | Österreichisches Dialyse- und Transplantationsregister (ÖDTR) | Nephrology | Österreichischen Gesellschaft für Nephrologie | Professional Society | disease registry | epidemi- ological | <https://www.nephrologie.at/gesellschaft/oedtr> | <https://doi.org/10.1159/000339102> |
| 58 | PRT - Prothesenregister Tirol | Surgery | LIV - Landesinstitut für Integrierte Versorgung Tirol | Hospital (Association) | intervention registry | quality assurance | <https://www.iet.at/page.cfm?vpath=register/prothesenregister> | https://www.iet.at/data.cfm?vpath=publikationen210/prt/euregio-bericht_2013-17_deu |
| 59 | Elektronischer Impfpass | General | ELGA GmbH | Government Organisation | intervention registry | adminis-trative | https://www.oesterreich.gv.at/themen/gesundheit_und_notfaelle/elektronischer_impfpass.html |  |
| 60 | Register der anzeigepflichtigen Krankheiten - Epidemiologisches Meldesystem | Infections | Agentur für Gesundheit und Ernährungssicherheit (AGES) | Government Organisation | disease registry | epidemi- ological | <https://covid19-dashboard.ages.at/basisinfo.html> |  |
| 61 | Register für hospitalisierte COVID19-Patientinnen und -Patienten | Infections | Gesundheit Österreich GmbH | Government Organisation | disease registry | epidemi- ological | <https://www.ris.bka.gv.at/Dokumente/BgblAuth/BGBLA_2022_II_26/BGBLA_2022_II_26.pdfsig> |  |
| 62 | Implantatregisters für den Bereich der Hüftendoprothetik | Implants | Gesundheit Österreich GmbH | Government Organisation | product registry | quality assurance | <https://www.sozialministerium.at/dam/jcr:a32545b2-d40c-43f2-ac1f-7c6ce97804a8/endoprothetik-bericht_27.07.18_final.pdf> |  |
| 63 | IVF-Register | Repro-duction | Gesundheit Österreich GmbH | Government Organisation | patient registry | quality assurance | <https://goeg.at/IVF-Reg> |  |
| 64 | Österreichisches Aortenklappenregister ÖAKlaR | Cardiology | Österreichischen Kardiologischen Gesellschaft Österreichische Gesellschaft für Thorax- und Herzchirurgie (ÖGTHC) | Professional Society | patient registry | quality assurance | <https://goeg.at/Aortenklappenregister> |  |
| 65 | Register für Screeningprogramme | Infections | Bundesministerium für Soziales, Gesundheit, Pflege und Konsumentenschutz | Government Organisation | disease registry | epidemi- ological | <https://www.ris.bka.gv.at/eli/bgbl/1950/186/P5b/NOR40223147> |  |
| 66 | AGO 16 IP-Register | Cancer | AGO Austria Arbeitsgemeinschaft für Gynäkologische Onkologie der OEGGG | Professional Society | intervention registry | epidemi- ological | <https://ago-austria.at/geschlossene-studien/> |  |
| 67 | AGO R02 – Brustrekonstruktion nach Mastektomie | Cancer | AGO Austria Arbeitsgemeinschaft für Gynäkologische Onkologie der OEGGG | Professional Society | intervention registry | epidemi- ological | <https://ago-austria.at/aktuelle-ago-studien/> |  |
| 68 | Datenhaltung Brustkrebs-Früherkennungsprogramm | Cancer | Gesundheit Österreich GmbH | Government Organisation | patient registry | epidemi- ological | <https://goeg.at/Datenhaltung_BKF> |  |
| 69 | Frühgeborenenregister | Birth | Gesundheit Österreich GmbH | Government Organisation | patient registry | quality assurance | <https://salk.at/25170.html> |  |
| 70 | Österreichisches Nachsorgeprogramm von Organ- und Stammzell-Lebendspender/innen | Trans-plantation | Gesundheit Österreich GmbH | Government Organisation | patient registry | clinical | <https://transplant.goeg.at/lebendspende> |  |
| 71 | Nationale Referenzzentrale für Meningokokken, Pneumokokken und Haemophilus influenzae | Infections | Agentur für Gesundheit und Ernährungssicherheit (AGES) | Government Organisation | disease registry | epidemi- ological | <https://www.ages.at/ages/referenzzentralen-labors/nationale-referenzzentrale-fuer-meningokokken-pneumokokken-und-haemophilus-influenzae> |  |
| 72 | ELGA e-Medikation | Pharma-ceutics | ELGA GmbH | Government Organisation | health care data base | adminis-trative | <https://www.elga.gv.at/> |  |
| 73 | AGMT AML Registry | Cancer | Arbeitsgemeinschaft medikamentöse Tumortherapie (AGMT) | Expert community | biobank | research | [https://www.agmt.at](https://www.agmt.at/) | 10.3390/ijms18020415  <https://doi.org/10.3390/cancers15051388>  <https://doi.org/10.1016/s2352-3026(22)00252-6>  <https://doi.org/10.3390%2Fcancers14102459>  <https://doi.org/10.1016/s2352-3026(20)30374-4>  <https://doi.org/10.1080/10428194.2017.1365854>  <https://doi.org/10.1007/s00508-018-1315-2>  <https://doi.org/10.1016/j.leukres.2017.05.006>  <https://doi.org/10.3390/ijms18040837>  <https://doi.org/10.3390/ijms18020415>  <https://doi.org/10.1186%2Fs13045-016-0263-4>  <https://doi.org/10.1016/j.leukres.2014.12.013>  <https://doi.org/10.1007/s00277-014-2126-9>  <https://doi.org/10.1016/j.leukres.2014.01.006>  <https://doi.org/10.1186%2F1756-8722-6-32> |

**Table 1b.** List of verified RWD Sources in Austria.

## Supplementary Table B. List of unverifiable lost RWD sources.

| **ID** | **Name of RWD** | **Description and further information of de-selection** |
| --- | --- | --- |
| 74 | AGMT Neck Cancer Registry | Registry not findable on <https://www.agmt.at/register/> any longer |
| 75 | Bone and soft tissue tumor registry | Other than the entry on orpha, there is no evidence that the register exists <http://www.orpha.net/data/> prj/AT/ID127487ger.pdf; |
| 76 | CEDATA-GPGE® Registry | Registry held and managed in Germany |
| 77 | ECFS Patient Registry | Registry held and managed not in Austria |
| 78 | European LeukemiaNet | Registry held and managed not in Austria |
| 79 | European Myelodysplastic Syndromes (EUMDS) Registry | Registry held and managed not in Austria |
| 80 | European Registry for Endocrine Surgery (Eurocrine®) | No Austrian data captured, Eurocrine is registered as a not for profit organisation organized and duly registered under the laws of Austria for societies. The owner of the platform is Region Skåne, the County Council of Scania Region in Sweden. |
| 81 | NF-10 - Prospective collection of potentially prognostically relevant data in patients with indolent non-follicular B-cell lymphoma | Not verifiable via 2nd source |
| 82 | Austrian registry for BRCA-1 and BRCA-2 mutation | Not verifiable via 2nd source |
| 83 | Medical claims database Austrian Ministry of Health | unspecific information about the real data source |
| 84 | Österreichisches Gesundheitsinformationssystem | No RWD for research purposes, only for monitoring and reporting. |
| 85 | Paediatric Congenital adrenal hyperplasia (CAH) registry | Registry held and managed not in Austria |
| 86 | REGIS – Regionales Gesundheitsinformationssystem | No RWD for research purposes, only for monitoring and reporting. |
| 87 | NSCLC Stadium III - Zentrales Datenregister für das Management von Patienten mit nichtkleinzelligem Lungenkrebs in Stadium III | unspecific information about the real data source. No further information is available apart from information on submission to the Ethics Committee of the Medical University of Vienna. |
| 88 | Österreichisches Register für fortgeschrittenes Prostata Karzinom | Not findable via online search |
| 89 | Peritonealkarzinose- Register der Medizinischen Universität Wien | Not findable via online search |
| 90 | Prospektives Register, mit Errichtung einer Biobank, und Genanalysen von Patienten mit Pulmonaler Hypertension | Not findable via online search |
| 91 | Register über die Behandlung von PatientInnen mit hirneigenen Tumoren an der KIM1 | Not findable via online search |
| 92 | AGO R01 Breast Cancer in Pregnancy Register Study (BCP) Registerstudie | Registry study, unclear whether own real-world data source |
| 93 | AGO R03 - ROC Register to Describe the Treatment Pattern of Platinum-sensitive Relapsed Epithelial Ovarian Cancer Patients in Austria | Registry study, unclear whether own real-world data source |
| 94 | AGO R05 AXillary Surgery After NeoAdjuvant Treatment (AXSANA) | Registry study, unclear whether own real-world data source |
| 95 | Observational study of pediatric thrombotic disease: the Throm-PED registry | Observational study, out of scope of RWD definition described in methods |
| 96 | Registry study in NSCLC patients with EGFR, ALK, or ROS1 mutations | Registry study, unclear whether own real-world data source |
| 97 | Covid-19 Datenplattform | No own data source |
| 98 | Styrian registry of congenital anomalies - contributes to the EUROCAT network | European Registry with Austrian Data https://eu-rd-platform.jrc.ec.europa.eu/sites/default/files/eurocat-pub-docs/JA%20EUROCAT%20Final%20Report.pdf |
| 99 | Non-Interventional, web-based Registry for Histiocytic Disorders | Such a register is not specified on the OeGHO homepage. |
| 100 | Akut PTCA (Perkutane Transluminale Coronare Angioplasie) Register der ÖKG | Website can no longer be accessed https://ptca.i-med.ac.at/ |
| 101 | Registry of the NHL-BFM study group for all subtypes of Non-Hodgkin lymphoma in children and adolescents | Not findable via online search |
| 102 | Registry for relapsing acute lymphoblastic leukemia in childhood and adolescence | Not findable via online search |
| 103 | Registry for Philadelphia chromosome-positive acute lymphoblastic leukemia in childhood and adolescence | Not findable via online search |
| 104 | Austrian Breast Implant Registry | 2009-2022: discontinued by founding professional society (Österreichische Gesellschaft für Plastische, Ästhetische und Rekonstruktive Chirurgie (ÖGPÄRC) |

**Table 2.** List of unverifiable lost RWD sources.

## Supplemtary Table C. RWD main purpose and type of data holder matrix.

|  | **Administrative** | **Clinical** | **Epidemiological** | **Quality assurance** | **Regulatory** | **Research** | **Total** |
| --- | --- | --- | --- | --- | --- | --- | --- |
| Expert community |  | 7 | 6 | 3 |  | 1 | **17** |
| Government Organisation | 6 | 2 | 7 | 6 | 6 |  | **27** |
| Hospital (Association) |  | 2 | 3 | 4 |  |  | **9** |
| Other |  |  |  | 1 |  |  | **1** |
| Professional Society |  | 1 | 5 | 4 |  |  | **10** |
| Social Insurance Institution |  |  |  | 1 |  |  | **1** |
| University |  | 3 | 3 |  |  | 2 | **8** |
| **Total** | **6** | **15** | **24** | **19** | **6** | **3** | **73** |

**Table 3.** RWD main purpose and type of data holder matrix.

## Supplementary Table D. Identified RWD holder.

| **No.** | **Name** |
| --- | --- |
| 1 | Agentur für Gesundheit und Ernährungssicherheit (AGES) |
| 2 | AGO Austria Arbeitsgemeinschaft für Gynäkologische Onkologie der OEGGG |
| 3 | Arbeitsgemeinschaft medikamentöse Tumortherapie (AGMT) |
| 4 | Arbeitskreis für Vorsorge- und Sozialmedizin |
| 5 | Austrian Mesothelioma Interest Group |
| 6 | BBMRI.at |
| 7 | Bundesamt für Sicherheit im Gesundheitswesen |
| 8 | Bundesministerium für Soziales, Gesundheit, Pflege und Konsumentenschutz |
| 9 | ELGA GmbH |
| 10 | Gesundheit Österreich GmbH |
| 11 | Klinikum Klagenfurt |
| 12 | LIV - Landesinstitut für Integrierte Versorgung Tirol |
| 13 | Medizinische Universität Graz |
| 14 | Medizinische Universität Innsbruck |
| 15 | Medizinische Universität Wien |
| 16 | Niederösterreichischen Landeskliniken-Holding |
| 17 | Österreichische Arbeitsgemeinschaft für Urogynäkologie & Rekonstruktive Beckenbodenchirurgie |
| 18 | Österreichische Gesellschaft für Chirurgie - OEGCH |
| 19 | Österreichische Gesellschaft für Hämatologie & Medizinische Onkologie |
| 20 | Österreichische Gesellschaft für Neurologie |
| 21 | Österreichische Hämophilie Gesellschaft |
| 22 | Österreichische Parkinsongesellschaft |
| 23 | Österreichische Sozialversicherung |
| 24 | Österreichischen Gesellschaft für Nephrologie |
| 25 | Österreichischen Kardiologischen Gesellschaft |
| 26 | Österreichischen Kardiologischen Gesellschaft Österreichische Gesellschaft für Thorax- und Herzchirurgie (ÖGTHC) |
| 27 | Statistik Austria |
| 28 | Trägerverein für das Österreichische Register für Biologica, Biosimilars und tsDMARDs bei der Behandlung von entzündlichen rheumatischen Erkrankungen (BioReg) |
| 29 | Tumorzentrum Oberösterreich |
| 30 | Uniklinikum Salzburg |

**Table 4.** Identified RWD holder.

## Supplementary Table E. Checklist on Quality criteria for RWD revised version 2.0.

| **Data management and stewardship** | - The "FAIR Data Principles" formulate principles that sustainable, reusable research data and research data infrastructures must meet. Definitions see here: <https://www.go-fair.org/fair-principles/> |
| --- | --- |
| **Governance framework** | - Available policy for collaborations with external organizations - Governance structure for decision-making on requests for collaboration - Available templates for research/data-sharing contracts - Involvement of Patient Organizations |
| **Quality requirements** | - High RWD quality standards are implemented – such as: completeness – accuracy – timeliness – comparability - Process in place for ongoing data quality assessments - Processes in place for quality planning, control, assurance and improvement - Data verification (method and frequency of verification) - Auditing practice |
| **Data privacy & transparency** | - Informed consent form and its validity for research purposes according to GDPR, EHDS and relevant national regulations. |
| **Research objectives** | *Note – Only applicable if the primary purpose of the RWD is research*   - Well defined research question outlined in a research plan - Available documentation, protocol or proposal which describes purpose of RWD use and rational that the RWD data sources adequately addresses the research questions (e.g. study protocol) - Approval of RWD use of independent review board/ethics committee - Protocol should follow the Declaration of Helsinki and furthermore the Declaration of Taipei [26] on Research on Health Databases, Big Data and Biobanks should be taken into account. |
| **Data providers** | - Description of data providers, such as patients, carers or health care professionals, their geographical area and any selection process (inclusion and exclusion criteria) that may be applied for their acceptance as data providers |
| **Patient population covered** | - Description of the type of patient population (disease, condition, time period covered, procedure), which defines the criteria for patient eligibility - Relevance of setting and catchment area - Clarity on patients’ inclusion and exclusion criteria - Methods applied to minimise selection bias and loss to follow-up |
| **Data elements** | - Definition, dictionary and format of data elements - Standards and terminologies applied - Capabilities and plans for amendments of data elements |
| **Infrastructure** | - High quality systems for RWD collection, recording and reporting, including timelines - Capability (and experience) for expedited reporting and evaluation of severe suspected adverse reactions in RWD collection - Capability (and experience) for periodic reporting of clinical outcomes – ideally patient reported outcomes - and adverse events reported by physicians, at individual-patient level and aggregated data level - Capability (and experience) for data cleaning, extraction, transformation and analysis - Capability (and experience) for data transfer to external organisations - Capabilities for amendment of safety reporting processes |

**Table 5.** Checklist on Quality criteria for RWD revised version 2.0.
